# ^18^F-FDG—PET/CT in Canine Mammary Gland Tumors

**DOI:** 10.3389/fvets.2019.00280

**Published:** 2019-08-27

**Authors:** Diana Sánchez, Laura Romero, Sergio López, Margarita Campuzano, Rocio Ortega, Alfonso Morales, Marina Guadarrama, Gabriela Cesarman-Maus, Osvaldo García-Pérez, Marcela Lizano

**Affiliations:** ^1^Unidad de Investigación Biomédica en Cáncer, Instituto Nacional de Cancerología–Instituto de Investigaciones Biomédicas, Universidad Nacional Autónoma de México, Mexico City, Mexico; ^2^Departamento de Medicina Genòmica y Toxicología Ambiental, Instituto de Investigaciones Biomédicas, Universidad Nacional Autónoma de México (UNAM), Mexico City, Mexico; ^3^Departmento de Patología, Facultad de Medicina Veterinaria y Zootecnia, Universidad Nacional Autónoma de México, Mexico City, Mexico; ^4^Departamento de Medicina Nuclear e Imagen Molecular, Instituto Nacional de Cancerología, Mexico City, Mexico; ^5^Departamento de Cirugía y Anestesia, Waldorf Pet Hospital, Mexico City, Mexico; ^6^Departamento de Cirugía y Anestesia, Hospital Kiin, Mexico City, Mexico; ^7^Departmento de Hematología, Instituto Nacional de Cancerología, Mexico City, Mexico

**Keywords:** canine mammary gland tumors, PET/CT, ^18^F-FDG, molecular imaging, cancer diagnosis

## Abstract

Medical imaging techniques play a central role in clinical oncology, helping to obtain important information about the extent of disease, and plan treatment. Advanced imaging modalities such as Positron Emission Tomography–Computed Tomography (PET/CT), may help in the whole-body staging in a single procedure, although the lesions should be carefully interpreted. PET/CT is becoming commonly used in canine cancer patients, but there is still limited information available on specific tumors such as mammary cancer. We evaluated the utility of fluorine-18 fluorodeoxyglucose (^18^F-FDG)-PET/CT to detect malignant lesions in eight female dogs with naturally occurring mammary tumors. A whole-body scan was performed prior to surgery, and mammary and non-mammary lesions detected either on PET/CT or during pre-surgical physical exam were resected when possible and submitted for histopathological examination. Multiple mammary lesions involving different mammary glands were detected in 5/8 dogs, for a total of 23 lesions; there were 11 non-mammary-located lesions in 6/8 dogs, three of these were lung or lymph node metastasis. A total of 34 lesions were analyzed: 22 malignant (19 mammary tumors and three metastatic lesions), and 12 benign (four mammary lesions and eight of non-mammary tissues). Glucose uptake by maximum standardized uptake value (SUVmax) was analyzed and correlated with tumor size, and benign vs. malignant pathology. We found that the minimum tumor size needed to distinguish malignant lesions according to the SUVmax was 1.5 cm; benign and malignant lesions <1.5 cm did not differ in glucose uptake (mean SUVmax = 1.1). In addition, a SUVmax value >2 was 100% sensitive for malignancy. Combining these data, lesions >1.5 cm with a SUVmax >2 had a positive predictive value of 100%. Finally, we did not find an association between SUVmax and histologic subtype or grade, which may be present in a larger sample. Thus, ^18^F-FDG PET/CT is useful for distinguishing malignant from benign lesion but further imaging of dogs with diverse tumors, should establish characteristic SUV value cutoffs for detecting primary and metastatic disease, and distinguishing them from benign lesions.

## Introduction

Dogs with mammary gland tumors are typically staged using fine needle aspiration of the primary tumor site and regional lymph nodes, as well as three-view chest radiographs. Excisional biopsies with histopathology and abdominal ultrasound are not routinely performed prior to surgery, although the former is necessary for a definitive diagnosis (1–3). The purpose of staging is to evaluate the extent of the disease (4). Results from staging assessment provide the basis for treatment planning, and give important prognostic information which may affect the owner's decision to consent to treatment (5). Advanced imaging modalities such as positron emission tomography/computed tomography (PET/CT), provide a way of achieving whole-body staging in a single imaging procedure (6, 7). This advanced nuclear imaging technique provides a good anatomic depiction of patients with tumor-related qualitative and quantitative metabolic information (8, 9). Because of the high glucose metabolic rates in most cancer cells, fluorine-18 fluorodeoxyglucose (^**18**^F-FDG) has been used as one radiotracer in PET/CT scans in oncology (10, 11). In comparison to normal cells, which metabolize glucose to generate energy mainly through oxidative phosphorylation, cancer cells resort to the use of aerobic glycolysis (Warburg phenomenon) as a means of generating ATP, thereby facilitating the incorporation of nutrients essential for cell proliferation into biomass (12, 13). In addition, cancer cells present an increased activity of glycolytic enzymes and glucose transporters, favoring glucose uptake (14, 15)]. ^**18**^F-FDG is a glucose analog in which the hydroxyl group on the two-carbon of a glucose molecule is replaced by a radioactive fluoride isotope (^**18**^F). FDG is taken up into living cells by facilitated transport and then phosphorylated by hexokinase. However, when FDG is fluorinated to form ^**18**^F-FDG, it cannot undergo further metabolism because the hydroxyl group at the two-carbon is required for this process (11). Cancer tissues present a higher uptake of ^**18**^F-FDG than normal tissues, due to their increased activity in glycolytic enzymes. Tracer accumulation provides information about tissue metabolic activity in terms of regional glucose uptake (PET), while the computed tomography provides exclusively the anatomic information (9, 16). PET/CT imaging in canine cancer patients has the potential to advance the current standard of care in veterinary oncology practice, but there is still scant information available characterizing the use of PET/CT in tumor-bearing dogs (17). In this study, we explore the utility of ^**18**^F-FDG PET/CT in canine mammary gland tumors.

## Materials and Methods

### Patients

Eight female dogs scheduled for mammary surgery were included in this study. All patients had at least one mammary gland lesion ≥0.5 cm of longest diameter and were programmed for a PET/CT scan 3 days before the surgical procedure. Mammary gland tumors as well as additional lesions detected either on PET/CT scan or during the pre-surgical physical exam were resected when possible and submitted for histopathological examination. Surgical tumor resection was performed according to standard practice. The type of surgery (lumpectomy, simple mastectomy, regional mastectomy or radical mastectomy) depended on the number of lesions, location, clinical stage, and owner's decision. Only lesions assessed by both PET/CT and post-surgical histopathology were included in this study. A total of 34 lesions were evaluated: 22 malignant and 12 benign. All patients were attended at the Hospital Veterinario de la Ciudad de México (HV-CDMX). This study was approved by an internal medical board of the HV-CDMX and carried out under owner's informed consent.

### ^18^F-FDG-PET/CT Imaging

Scans were carried out using a Siemens PET/CT Biograph 16 (Siemens Healthcare, Erlangen, Germany), with patients under sedation with dexmedetomidine 0.375 mcg/m^2^ IV, plus maintenance fluid therapy and oxygen supplementation with mask (0.5-1L/Kg). Patients were rested and fasted 8–12 h before the injection of 0.2 mCi/Kg (7.4 MBq) of ^18^F-FDG. Before ^18^F-FDG administration, serum glucose levels were assessed; all dog had levels within normal reference values. After injection, patients were rested 25–45 min in the dark and at a room temperature. Immediately before CT acquisition, dogs were anesthetized and iodide contrast was injected (0.4 ml/Kg of iopamidol, intravenously). CT images were performed immediately before PET scanning using the multi-detector four-slice spiral CT scanner, and acquired as contrast-enhanced CT scans. The CT scan was carried out with a rotation time of 0.5 s, a speed of 16 mm per rotation, helical thickness 4 mm, pitch 0.5 with 120 kV and 150 mA. The PET scan followed immediately with an acquisition time of 3 min per bed position and reconstructed using 4 iterations and 14 Subsets, a Butterworth filter with 5 mm FWHM was used. Whole-body PET scanning consisted of imaging from the nose to the tail using four to eight axial fields of view with coverage of 14 cm. CT data were used for attenuation correction of the PET data. Both image sets were reconstructed in trans-axial, coronal, and sagittal images with a slice thickness of 4 mm.

### Image Analysis

Three nuclear medicine physicians in collaboration interpreted the images on the Siemens Syngo Via Workstation (S.L., M.C., and O.G.). For each study, a large region of interest (ROI) was drawn manually around the entire primary tumor, and maximum standardized uptake values (SUVmax) were obtained for further analyses.

### Histopathology

The formalin-fixed paraffin-embedded tissues were cut serially into 4 μm sections and stained with hematoxylin and eosin (H&E). A veterinary pathologist (L.R.) classified all lesions. Histopathological description included diagnosis of tumor-type and grading (18).

### Statistical Analysis

To determine whether there was an association between clinical characteristics, malignancy, and glucose uptake, we examined the following variables: number of tumors, anatomic tumor site, tumor size, benign vs. malignant histology and histologic subtype and grade. Continuous data was expressed as mean ± standard deviation (SD). To assess differences between variables, one-way ANOVA and Turkey's multiple comparison test or unpaired *t*-test were used. Spearman's rank correlation test was used to analyze the linear relationship for quantitative data. For sensitivity, specificity, and predictive values Chi-square and Fisher's exact tests were used. Significance was set at *p* < 0.05. All analyses were performed using Prisma statistical software version 6.0.

## Results

### Patient Characteristics

By the time of surgery, 4/8 female dogs were spayed (50%) and four were intact (50%); the age range was 7–15 years; three patients (37.5%) had only benign mammary lesions while the other five (62.5%) had at least one malignant mammary gland tumor. Clinical stages were I:1 (dog 7); II:1 (dog 5); IV:2 (dogs 2 and 4) and V:1 (dog 1). During surgery, intact dogs were spayed. In addition to mammary surgery, in four patients an additional procedure was performed including partial lung lobectomy, resection of cutaneous lesions, popliteal lymph node biopsy, and umbilical herniorrhaphy (Table 1).

**Table 1:** Clinical characteristics of the 8 female patients with mammary gland tumors evaluated by PET/CT.

**Patient ID**	**Breed**	**Castration status**	**Age (years)**	**No. of Mammary Gland Tumors**		Clinical stage^*^	**Surgery/Relevant clinic history**
				**Benign**	**Malignant**		
1	Miniature Schnauzer	Intact	15	0	4	V	Partial lung lobectomy, oophorectomy/hysterectomy, regional mastectomy, and lumpectomy. Previous resection of mammary tumors (1 year before).
2	Poodle-mix	Intact	14	0	8	IV	Left radical mastectomy, right regional mastectomy, and oophorectomy/hysterectomy.
3	Mix breed	Spayed	12	2	0	NA	Left regional mastectomy and resection of cutaneous lesions. Previous history of mammary tumors, mitral valve disease.
4	Poodle-mix	Spayed	7	0	1	IV	Right regional mastectomy and 2 CT cycles with 5FU/CTX / Previous CT for TVT.
5	Terrier-mix	Spayed	14	0	2	II	Bilateral regional mastectomy.
6	Golden retriever	Intact	8	1	0	NA	Right regional mastectomy and oophorectomy/hysterectomy.
7	Mix breed	Intact	9	0	4	I	Bilateral radical mastectomy, oophorectomy/hysterectomy, resection of cutaneous lesion, and biopsy of a popliteal lymph node.
8	Mix breed	Spayed	7	1	0	NA	Right regional mastectomy, umbilical herniorrhaphy.

* Considering the size of the larger tumor.

*NA, Does not apply; 5FU, 5-fluorouracil; CTX, cyclophosphamide; CT, chemotherapy; TVT, transmissible venereal tumor*.

**Figure 1:**
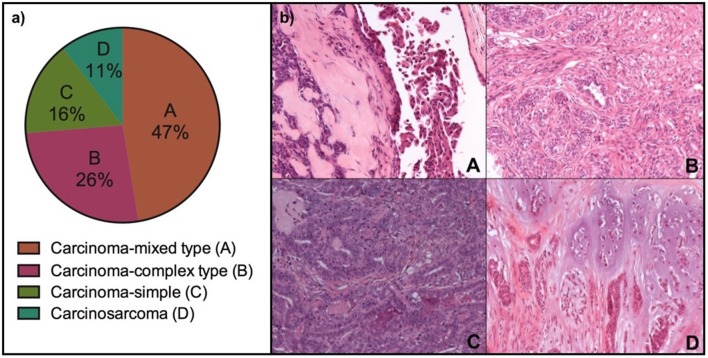
Histological subtypes of malignant mammary gland tumors analyzed by PET/CT in eight female dogs. **(a)** Percentage of tumors by histologic subtype; **(b)** Representative photomicrographs of the four histologic subtypes by HandE staining, 20x (A, Carcinoma-mixed type; B, Carcinoma-complex type; C, Carcinoma-simple; and D, Carcinosarcoma).

A total of 34 lesions were resected and analyzed by histopathology: 23 mammary gland tumors and 11 located at other anatomical sites. The mammary lesions ranged between 0.3 and 6.1 cm and were most frequently detected in the caudal-abdominal and inguinal mammary glands (16/23, 70%), while thoracic and cranial-abdominal mammary glands together were affected in only 30% of cases (7/23). Most (83%, 19/23) mammary lesions were malignant. The malignant lesions (*n* = 19) were diagnosed as carcinoma-mixed type (9/19), carcinoma-complex type (5/19), carcinoma-simple (3/19), and carcinosarcoma (2/19). Near half (47%, 9/19) of malignant tumors were represented by mixed carcinomas (Table 2, Figure 1).

**Table 2:** Mammary gland tumors evaluated by ^18^F-FDG PET/CT: Tumor size (cm), type (benign vs. malignant), and location (mammary vs. extrammamary).

**Mammary gland**	**1**		**2**		**3**		**4**		**5**		**Total of lesions**		**Additional lesions resected**
	**R**	**L**	**R**	**L**	**R**	**L**	**R**	**L**	**R**	**L**	**B**	**M**	
1	–	M 0.6 cm	–	–	–	M 0.7 cm	M 0.8 cm	–	–	M 3.2 cm	0	4	2: Lung metastasis (1.7 cm) and endometrial hyperplasia (1.9 cm).
2	–	M 3.0 cm	M 0.5 cm	M 1.0 cm	–	M 0.5 cm	M 0.3 cm	M 2.0 cm	M 0.5 cm	M 2.0 cm	0	8	2: Endometrial hyperplasia, and metastatic lymph node (0.2 cm).
3	–	–	–	–	–	–	B 0.5 cm	–	–	B 0.5 cm	2	0	3: Cutaneous melanocytoma (0.5 cm) and two benign skin lesions (0.5 cm both).
4	–	–	–	–	–	–	M 0.5 cm	–	–	–	0	1	1: Malignant lymph node (0.4 cm).
5	–	–	–	–	–	–	M 3.2 cm	M 2.3 cm	–	–	0	2	–
6	–	–	–	–	B 6.1 cm	–	–	–	–	–	1	0	1: Benign adipose tissue.
7	–	–	–	–	–	–	M 2.5 cm	M 0.7 cm	M 1.5 cm	M 0.8 cm	0	4	2: Cutaneous hemangioma (2.7 cm) and benign lymph node (1 cm).
8	–	–	–	–	–	–	B 1.7 cm	–	–	–	1	0	–
Total	0	2	1	1	1	2	7	3	2	4	4	19	11
	7						16				23		11
	23												
	34												

*Mammary gland 1–5: cranial-thoracic, caudal-thoracic, cranial-abdominal, caudal-abdominal and inguinal, respectively; R, right; L, left; M, malignant; B, benign*.

Regarding non-mammary gland lesions (11), 73% were benign (8/11) and include: endometrial hyperplasia, lipoma, and cutaneous lesions. The remaining 27% (3/11) corresponded to malignant lymph nodes and a lung metastasis.

### ^18^F-FDG-PET/CT Findings

#### Cancer Patients

**Figure 2:**
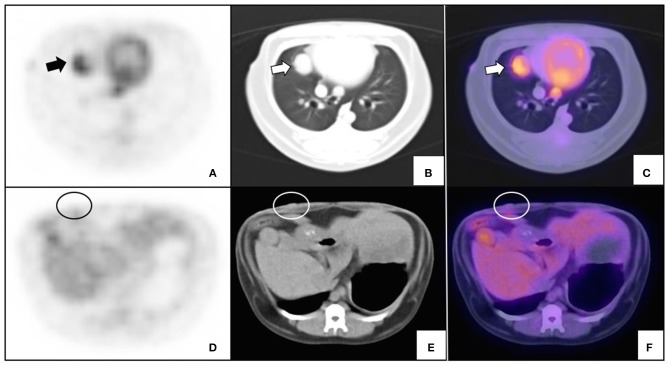
Variable glucose uptake of lung metastasis and primary malignant mammary tumor. An intact 15-year old female miniature Schnauzer with mammary cancer metastatic to lung. **(A–C)** Transverse slice of histopathologically confirmed lung metastasis (arrows); and **(D–F)** confirmed mammary cancer (circle). PET **(A,D)**, CT **(B,E)**, and fused imaging **(C,F)** are shown. SUVmax for lung metastasis was 2.14 and <0.7 for the smaller mammary tumor.

Five of eight patients were confirmed to have malignant mammary tumors. One of them had a single tumor, while the others had multiple tumors with different histologic subtype, grade, and SUVmax values. The SUVmax between individual lesions per dog ranged between 0.56 and 0.67 (Dog 1); 0.71–3.17 (Dog 2); 1.23 (Dog 4, single lesion); 2.07–2.45 (Dog 5), and 1.06-3.01 (Dog 7) in each patient, respectively. Three dogs had advanced disease stage IV (two with metastatic lymph nodes), and V (one dog with lung metastasis). The SUVmax for the metastatic lesions was 0.56 and 0.84 for metastatic lymph nodes and 2.14 for the lung metastasis. The lung metastasis showed a 3-fold increased SUVmax compared with the smaller mammary tumors of the same dog (Figure 2; Table 3). This stage V patient had history of a previous resection of a mammary tumor (without histopathological analysis), as well as two other ^18^F-FDG-positive lung lesions clinically considered as metastasis, according to the natural history of the disease, although not considered for the statistics because they were not confirmed by histopathology, so they did not meet the inclusion criteria.

**Table 3:** Patients with malignant mammary tumors.

**Mammary gland**	**1**		**2**		**3**		**4**		**5**		**Metastatic lesions**
	**R**	**L**	**R**	**L**	**R**	**L**	**R**	**L**	**R**	**L**	
1	–	0.6 cm carcinoma-mixed type, 1 **SUV**_**max**_ **0.59**	–	–	–	0.7 cm Carcinoma-complex type, 1 **SUV**_**max**_ **0.59**	0.8 cmcarcinoma-mixed type, 1 **SUV**_**max**_ **0.56**	–	–	3.2 cm Carcinosarcoma, 2 **SUV**_**max**_ **0.67**	1.7 cm Lung metastasis **SUV**_**max**_ **2.14**
2	–	3.0 cm Carcinoma-mixed type, 1 **SUV**_**max**_ **2.12**	0.5 cm Carcinoma- mixed type, 1 **SUV**_**max**_ **1.01**	1.0 cmCarcinoma-simple, 1 **SUV**_**max**_ **0.77**	–	0.5 cm Carcinoma-complex type, 1 **SUV**_**max**_ **0.71**	0.3 cmCarcinoma-mixed type, 1 **SUV**_**max**_ **0.71**	2.0 cm Carcinoma-mixed type, 1**SUV**_**max**_ **3.17**	0.5 cm Carcinoma-simple, 3 **SUV**_**max**_ **2.2**	2.0 cm Carcinoma-simple, 1 **SUV**_**max**_ **2.4**	0.2 cm Lymph node **SUV**_**max**_ **0.84**
4	–	–	–	–	–	–	0.5 cm Carcinoma-mixed type, 3 **SUV**_**max**_ **1.23**	–	–	–	0.4 cm Lymph node **SUV**_**max**_ **0.56**
5	–	–	–	–	–	–	3.2 cm Carcinoma mixed-type, 2 **SUV**_**max**_ **2.45**	2.3 cm Carcinosarcoma, 2 **SUV**_**max**_ **2.07**	–	–	–
7	–	–	–	–	–	–	2.5 cm Carcinoma-mixed type, 2 **SUV**_**max**_ **3.01**	0.7 cm Carcinoma-complex type, 1 **SUV**_**max**_ **1.06**	1.5 cm Carcinoma-complex type, 1 **SUV**_**max**_ **1.7**	0.8 cmCarcinoma-complex type, 2 **SUV**_**max**_ **2.6**	–

*Tumor location, size, histologic subtype, grade and SUV_max_ values*.

*Mammary gland 1–5: cranial-thoracic, caudal-thoracic, cranial-abdominal, caudal-abdominal, and inguinal, respectively; R, right; L, left*.

*SUV_max_: maximum standardized uptake value*.

### Non-cancer Patients

**Figure 3:**
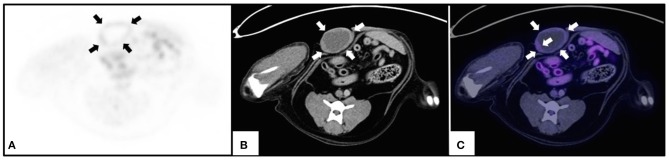
PET/CT of a benign canine mammary gland tumor. An intact female 8-year old, Golden retriever, with a 6.1 cm tumor of longest diameter on the right cranial-abdominal gland which corresponded to a fibroadenoma. The figure shows the same transverse slice by **(A)** PET; **(B)** CT, and **(C)** image fusion. Arrows indicate the periphery of the tumor and the increase in metabolic glucose uptake is shown in purple **(C)**.

Three dogs had only benign lesions ranging from 0.5 to 6.1 cm. One of the dogs had two small lesions (0.5 cm each one) corresponding to a benign mixed tumor (SUVmax 0.57) and a foreign body reaction (SUVmax 0.66), while the other two dogs had only one lesion that corresponded to a benign mixed tumor (1.7 cm, SUVmax 1.68) and a fibroadenoma (6.1 cm, SUVmax 1.96). The bigger tumor showed only in the peripheral glucose metabolism (Figure 3).

### Relation Between Tumor Size and ^18^F-FDG Uptake (SUVmax)

**Figure 4:**
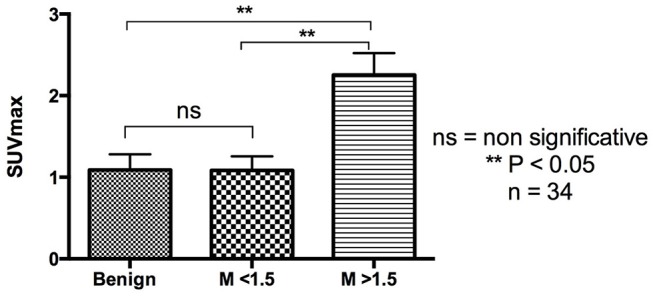
A minimum of 1.5 cm of tumor size is necessary to identify malignant mammary tumors according to their SUVmax. The graph shows results of an ordinary one-way ANOVA analysis for benign and malignant (M) lesions with a cut-off value of 1.5 cm.

**Figure 5:**
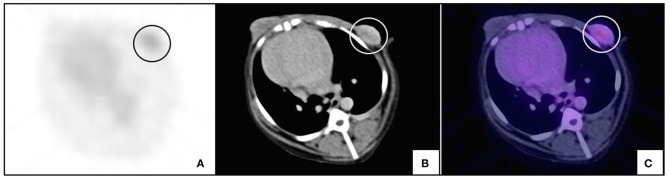
^18^F-FDG-PET/CT of a malignant canine mammary gland tumor. An intact 14-year old female Poodle-mix, with multiple mammary tumors. The figure shows the same transversal slice by **(A)** PET; **(B)** CT, and **(C)** fusion images of a 3 cm carcinoma-mixed type tumor (circle) (SUVmax: 2.12).

We analyzed the relation between size and ^18^F-FDG uptake (SUVmax) in the 32 lesions. Considering the previous data reported in human tumors (19, 20), we expected a correlation between both variables. In this study, a moderate positive correlation (*p* = 0.0017, *r* = 0.53) was observed between these two variables. We excluded two lesions for this analysis because of the absence of precise size documentation: an endometrial hyperplasia and one benign adipose tissue. To confirm this finding, it is necessary to evaluate the behavior of this correlation in a bigger sample. In the other hand, to determine whether at certain tumor size cutoff, the SUVmax could differentiate between malignant vs. benign lesions in out cohort, we analyzed all lesions (*n* = 34) using different cut-off values. The minimum size in which the SUVmax was useful to differentiate malignant vs. benign lesion in our data, was 1.5 cm (Figures 4, 5). However, in subsequent analyzes this cut off value as a single predictor of malignancy, had a sensitivity of 41% and specificity of 60%.

### A SUVmax >2 Is 100% Sensitive for Identifying Canine Malignant Mammary Gland Tumors Independently of Tumor Size

**Figure 6:**
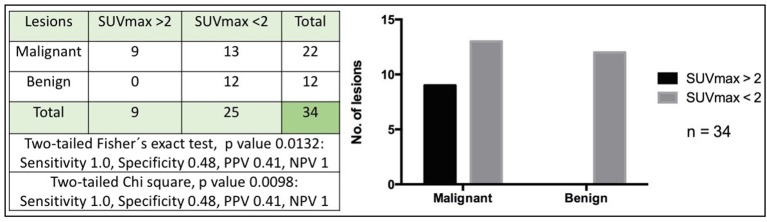
High sensitivity of SUVmax >2 for detecting canine malignant mammary tumors. The figure shows the number of malignant or benign lesions according to a cut-off SUVmax value of 2. A two-tailed Fisher's exact test and Chi-square were used (*p* < 0.05). PPV, Positive predictive value; NPV, Negative predictive value.

We analyzed all lesions to determine the SUVmax cutoff value where a specificity and negative predictive value were significant. In this study, we found that a SUVmax >2 had a sensitivity and a negative predictive value of 100% although with a specificity of 48%, and a positive predictive value of 41% (*p* < 0.05). This means that 100% of malignant tumors had a SUVmax>2 but only 49% of benign lesions had a SUVmax <2. In terms of predictive values, a SUVmax <2 had 100% probability of detecting benign lesions, while there was a 41% of probability that a lesion with a SUVmax >2 was malignant (Figure 6). Considering the small size of our sample, this value should be taken with caution and confirmed later. There could be aggressive histologic subtypes with low SUVmax values, as has been reported in human breast cancer (21).

### The Tumor Size Plus the SUVmax Value Could Accurately Identify Mammary Cancer

**Figure 7:**
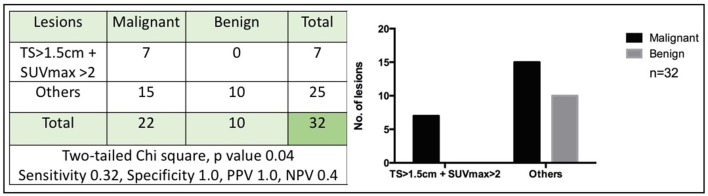
Tumor size >1.5 cm with SUVmax >2 accurately identifies mammary cancer. The figure shows the number of malignant or benign lesions according to a cut-off tumor size (TS) of 1.5 cm in conjunction with a cut-off SUVmax value of 2. A two-tailed Chi-square was used (*p* < 0.05). PPV, Positive predictive value; NPV, Negative predictive value.

Given that we found that both SUVmax and tumor size could help in identify malignant mammary lesions, we evaluated if their combination could improve the detection of mammary cancer. For this analysis we exclude two lesions because of the absence of precise size documentation: an endometrial hyperplasia and one benign adipose tissue tumor. We found that a tumor size >1.5 cm plus a SUVmax value >2 had a specificity and a positive predictive value of 100% as well as a 32% sensitivity and a negative predictive value of 40% (Figure 7). In our sample, both parameters in combination, accurately identified mammary cancer. Nevertheless, smaller tumors or lesions with SUVmax <2 does not rule out cancer (Figure 7). In addition, we evaluated if there was a correlation between SUVmax and histologic cancer subtype or grade but did not find a statistically significant association (one-way ANOVA *p* = 0.89 and *p* = 0.25, respectively).

## Discussion

The utility of ^18^F-FDG PET/CT has been demonstrated for both staging and evaluation of treatment response in canine cancer (17, 22). The imaging characteristics and clinical value for diverse types of cancer are still being investigated. SUVmax values using ^18^F-FDG have been reported for various tumors such as fibrosarcoma, adenocarcinoma, squamous cell carcinoma, soft tissue sarcoma, hemagiopericytoma, hemangiosarcoma, histiocytic sarcoma, lymphoma, primary lung tumors, Sertoli cell tumor and gastrointestinal stromal tumor, with values ranging between 2 and 27 (17, 22, 23). Carcinomas have been shown to have a higher SUVmax (7.6–27) than mesenchymal tumors (2–10.6) (22, 23).

Regarding mammary tumors, ^18^F-FDG-PET/CT studies have been reported in only four patients with mammary carcinomas. In these cases, SUVmax values in the primary tumors were all above two (range 2.97–10.01) (17) In our study we found that the SUVmax values above >2 had a high positive predictive value for malignancy. This data resembles observations from previous publications in human patients, in whom malignant breast tumors usually show SUVmax values >2 (24, 25). Furthermore, when we used a combination of both tumor size >1.5 cm and SUVmax >2, the specificity and positive predictive value reached 100%, meaning that mammary lesions that meet these two criteria were all corroborated to be malignant.

We also looked if histologic subtype, grade or anatomic site were associated with a characteristic range of SUVmax values and did not find any relevant differences. However, given that some malignant lesions were smaller or had a low glucose uptake, the absence of these two criteria did not discard cancer. Based on human experience where there is a correlation between SUVmax and histologic subtypes or grade, among others (26–28), and given the similarities between human breast cancer and canine mammary gland tumors (29), it is reasonable to expect an association between these parameters.

It is important to highlight that one limitation of our study is the sample size, which restricted the number and homogeneity of the evaluated groups, so the results should be taken with caution since they reflect only the studied sample. Additional studies should confirm the results. The effect of the reconstruction parameters of the image should be taken into account, such as the partial volume effect (not studied in this group of tumors). In small lesions, according to the partial resolution of the PET/CT scan used, the SUVmax value is affected and may cause quantification errors. In addition, the histologic subtype of tumors could affect the obtained result, as in humans some types of benign tumors are known to have high FDG accumulation and are therefore potential causes for false positive diagnoses. These tumors include fibrous mesothelioma, schwannoma, aggressive neurofibromas, and enchondromas (30). It would also be interesting to study in dogs, the value of ^18^F-FDG-PET/CT in relation to tumor molecular profiles.

In conclusion, molecular imaging techniques are being increasingly used in veterinary cancer patients. We found promising applicability of ^18^F-FDG PET/CT for the pre-surgical study of canine mammary gland tumors. It is clinically useful to establish standardized imaging protocols as well as characteristic glucose uptakes, and to establish imaging limitations on tumor size. Dogs typically have more than one lesion, thus the ability to stage patients and distinguish between benign and malignant lesions, previous to a surgery, should lead to better treatment planning and precise information which will affect the owner's decision to consent to treatment.

## Data Availability

The datasets generated for this study are available on request to the corresponding author.

## Author Contributions

DS, ML, GC-M, and OG-P contributed conception and design of the study. DS, LR, RO, AM, OG-P, SL, and MG acquired data. DS, LR, GC-M, ML, SL, MC, and OG-P organized, analyzed, and interpreted data. DS, GC-M, OG-P, and ML drafted and revised article for intellectual content. ML and OG-P read and final approved the completed article.

### Conflict of Interest Statement

The authors declare that the research was conducted in the absence of any commercial or financial relationships that could be construed as a potential conflict of interest.

## References

[1] Cassali GD Lavalle GE De Nardi AB Ferreira E Bertagnolli AC Estrela-Lima A Consensus for the diagnosis, prognosis and treatment of canine mammary tumors. Braz J Vet Pathol. (2011) 4:153–80.

[2] Biller B Berg J Garrett L Ruslander D Wearing R Abbott B. AAHA oncology guidelines for dogs and cats. J Am Anim Hosp Assoc. (2016) 52:181–204. 10.5326/JAAHA-MS-6570 27259020

[3] Gundim LF De Araujo CP Blanca WT Guimaraes EC Medeiros AA. Clinical staging in bitches with mammary tumors: Influence of type and histological grade. Can J Vet Res. (2016) 80:318–22. 27733787 PMC5052884

[4] Brierley J Gospodarowicz M O'sullivan B. The principles of cancer staging. Ecancermedicalscience. (2016) 10:ed61. 10.3332/ecancer.2016.ed61 28101141 PMC5215238

[5] Sorenmo K. Canine mammary gland tumors. Vet Clin North Am Small Anim Pract. (2003) 33:573–96. 10.1016/S0195-5616(03)00020-2 12852237

[6] National Research nd Institute of Medicine Committee on State of the Science of Nuclear M The National Academies Collection: reports funded by national institutes of health. In: Advancing Nuclear Medicine Through Innovation. Washington, DC: National Academies Press (2007).

[7] Specht JM Mankoff DA. Advances in molecular imaging for breast cancer detection and characterization. Breast Cancer Res. (2012) 14:206. 10.1186/bcr3094 22423895 PMC3446362

[8] Farwell MD Pryma DA Mankoff DA. PET/CT imaging in cancer: current applications and future directions. Cancer. (2014) 120:3433–45. 10.1002/cncr.28860 24947987

[9] Randall EK PET-computed tomography in veterinary medicine. Vet Clin North Am Small Anim Pract. (201 46:515–33, vi. 10.1016/j.cvsm.2015.12.008 27068445

[10] Agrawal A Rangarajan V. Appropriateness criteria of FDG PET/CT in oncology. Indian J Radiol Imaging. (2015) 25:88–101. 10.4103/0971-3026.155823 25969632 PMC4419439

[11] Shen B Huang T Sun Y Jin Z Li XF Revisit 18F-fluorodeoxyglucose oncology positron emission tomography: “systems molecular imaging” of glucose metabolism. Oncotarget. (2017) 8:43536–42. 10.18632/oncotarget.16647 28402949 PMC5522167

[12] Ganapathy V Thangaraju M Prasad PD. Nutrient transporters in cancer: relevance to Warburg hypothesis and beyond. Pharmacol Ther. (2009) 121:29–40. 10.1016/j.pharmthera.2008.09.005 18992769

[13] Vander Heiden MG Cantley LC Thompson CB. Understanding the Warburg effect: the metabolic requirements of cell proliferation. Science. (2009) 324:1029–33. 10.1126/science.1160809 19460998 PMC2849637

[14] Rodriguez-Enriquez S Marin-Hernandez A Gallardo-Perez JC Moreno-Sanchez R. Kinetics of transport and phosphorylation of glucose in cancer cells. J Cell Physiol. (2009) 221:552–9. 10.1002/jcp.21885 19681047

[15] Adekola K Rosen ST Shanmugam M. Glucose transporters in cancer metabolism. Curr Opin Oncol. (2012) 24:650–4. 10.1097/CCO.0b013e328356da72 22913968 PMC6392426

[16] Vercher-Conejero JL Pelegrí-Martinez L Lopez-Aznar D Cózar-Santiago MP. Positron emission tomography in breast cancer. Diagnostics. (2015) 5:61–83. 10.3390/diagnostics5010061 26854143 PMC4665546

[17] Seiler SM Baumgartner C Hirschberger J Beer AJ Bruhschwein A Kreutzmann N. Comparative oncology: evaluation of 2-Deoxy-2-[18F]fluoro-D-glucose (FDG) positron emission tomography/computed tomography (PET/CT) for the staging of dogs with malignant tumors. PLoS ONE. (2015) 10:e0127800. 10.1371/journal.pone.0127800 26068641 PMC4466332

[18] Goldschmidt M Pena L Rasotto R Zappulli V. Classification and grading of canine mammary tumors. Vet Pathol. (2011) 48:117–31. 10.1177/0300985810393258 21266722

[19] Jo I Zeon SK Kim SH Kim HW Kang SH Kwon SY. Correlation of primary tumor FDG uptake with clinicopathologic prognostic factors in invasive ductal carcinoma of the breast. Nucl Med Mol Imaging. (2015) 49:19–25. 10.1007/s13139-014-0296-y 25774234 PMC4354784

[20] Shen G Hu S Kuang A Relationship between FDG uptake on PET, tumor histology, and Ki-67 proliferation index in patients with breast cancer. J Nuclear Med. (2016) 57:1492–1492.

[21] Fujii T Yajima R Kurozumi S Higuchi T Obayashi S Tokiniwa H. Clinical significance of 18F-FDG-PET in invasive lobular carcinoma. Anticancer Res. (2016) 36:5481–5. 10.21873/anticanres.11129 27798919

[22] Borgatti A Winter AL Stuebner K Scott R Ober CP Anderson KL. Evaluation of 18-F-fluoro-2-deoxyglucose (FDG) positron emission tomography/computed tomography (PET/CT) as a staging and monitoring tool for dogs with stage-2 splenic hemangiosarcoma - A pilot study. PLoS ONE. (2017) 12:e0172651. 10.1371/journal.pone.0172651 28222142 PMC5319762

[23] Hansen AE Mcevoy F Engelholm SA Law I Kristensen AT. FDG PET/CT imaging in canine cancer patients. Vet Radiol Ultrasound. (2011) 52:201–6. 10.1111/j.1740-8261.2010.01757.x 21388475

[24] Sanli Y Kuyumcu S Ozkan ZG Isik G Karanlik H Guzelbey B. Increased FDG uptake in breast cancer is associated with prognostic factors. Ann Nucl Med. (2012) 26:345–50. 10.1007/s12149-012-0579-2 22359222

[25] Yararbas U Avci NC Yeniay L Argon AM. The value of 18F-FDG PET/CT imaging in breast cancer staging. Bosn J Basic Med Sci. (2018) 18:72–9. 10.17305/bjbms.2017.2179 28763628 PMC5826677

[26] Ekmekcioglu O Aliyev A Yilmaz S Arslan E Kaya R Kocael P. Correlation of 18F-fluorodeoxyglucose uptake with histopathological prognostic factors in breast carcinoma. Nucl Med Commun. (2013) 34:1055–67. 10.1097/MNM.0b013e3283658369 24025919

[27] Jung NY Kim SH Choi BB Kim SH Sung MS. Associations between the standardized uptake value of (18)F-FDG PET/CT and the prognostic factors of invasive lobular carcinoma: in comparison with invasive ductal carcinoma. World J Surg Oncol. (2015) 13:113. 10.1186/s12957-015-0522-9 25889560 PMC4371618

[28] Has Simsek D Sanli Y Kulle CB Karanlik H Kilic B Kuyumcu S. Correlation of 18F-FDG PET/CT with pathological features and survival in primary breast cancer. Nucl Med Commun. (2017) 38:694–700. 10.1097/MNM.0000000000000694 28557954

[29] Queiroga FL Raposo T Carvalho MI Prada J Pires I. Canine mammary tumours as a model to study human breast cancer: most recent findings. In Vivo. (2011) 25:455–65. 21576423

[30] Carter KR Kotlyarov E Common causes of false positive F18 FDG PET/CT scans in oncology. Braz Arch Biol Technol. (2007) 50:29–35. 10.1590/S1516-89132007000600004

